# Circulating hypoxia-dependent miR-210 is increased in clinical sepsis subtypes: A cohort study

**DOI:** 10.1186/s12967-022-03655-6

**Published:** 2022-10-04

**Authors:** Rachel E Powell, Yi Yin Tai, Jason N Kennedy, Christopher W Seymour, Stephen Y Chan

**Affiliations:** 1 Division of Pulmonary, Allergy, and Critical Care Medicine, University of Pittsburgh School of Medicine, Pittsburgh, PA USA; 2 Center for Pulmonary Vascular Biology and Medicine, Pittsburgh Heart, Lung, and Blood Vascular Medicine Institute, Division of Cardiology, Department of Medicine, University of Pittsburgh School of Medicine, 200 Lothrop Street BST E1240, 15261 Lung, Pittsburgh, PA USA; 3 Clinical Research Investigation and Systems Modeling of Acute Illness (CRISMA) Center, University of Pittsburgh School of Medicine, Pittsburgh, PA USA; 4 Department of Critical Care Medicine, University of Pittsburgh School of Medicine, Pittsburgh, PA USA; 5 Department of Emergency Medicine, University of Pittsburgh School of Medicine, Pittsburgh, PA USA

**Keywords:** Sepsis, miR-210, Hypoxia

## Dear editor,

Patients with community-onset sepsis can be grouped into 4 distinct subtypes (alpha, beta, gamma, and delta) based on routinely available clinical data [1]. These subtypes differ both in outcome and response to treatment, though the biologic mechanisms underlying these differences are unknown. Because ischemic end-organ damage and endothelial dysfunction may contribute to differences in sepsis [2], we hypothesize that treatment-response subtypes may be partially explained by differential activation of hypoxia-mediated pathways. Multiple studies show that circulating microRNAs (miRNAs) are candidate biomarkers for acute illness, but research in sepsis is limited [3]. As a ubiquitously expressed miRNA that regulates key downstream responses to hypoxia [4] and can be delivered to the endothelium as a biologically active molecule from the circulating bloodstream [5], miR-210 is a promising candidate to study in sepsis.

This study was approved by the University of Pittsburgh Human Research Protection Office. The data were obtained under a waiver of informed consent and with authorization under the Health Insurance Portability and Accountability Act. We used electronic health record data and plasma EDTA from adult patients meeting Sepsis-3 criteria within 6 h of presentation to a tertiary care center in southwestern Pennsylvania from 2017 to 2019. Clinical data was used to assign patients to one of four sepsis subtypes, using reproduced methods [1]. We randomly selected 20 patients from each sepsis subtype, and 20 non-septic controls for comparison from prior acute illness cohorts. We extracted circulating miRNA from plasma and quantified expression of miR-210 using qRT-PCR. Fold change (FC) was determined relative to the non-septic controls. One-way ANOVA and pairwise t-tests, with Tukey’s procedure to adjust for multiple comparisons, was used to compare fold change after log transformation. We used multivariable logistic regression to quantify the risk-adjusted association between miR-210 expression and the delta subtype. Model covariates were chosen a priori based on factors known to be associated with sepsis-specific outcomes, including age, sex, Elixhauser Comorbidity Index (range, 0–31), and presenting Sequential Organ Failure Assessment (SOFA; range, 0–24) score. Further details are provided in the Supplement.

Of 80 sepsis patients, 59 (74%) were white, 40 (50%) were male, and the median age was 62 (IQR: 52–73) years. Delta subtype patients had higher illness severity (median SOFA score 6 vs. 3, p < 0.001), higher rates of ICU admission (95 vs. 51%, p < 0.001), and more vasopressor use (55 vs. 12%, p < 0.001) than non-delta subtype patients. The expression of miR-210 was increased in all subtypes of sepsis compared to control (**Figure**), with a median 3.6-fold increase (p < 0.001). Compared to non-delta sepsis subtypes, expression of miR-210 was substantially increased in the plasma of patients with delta subtype (10.2-fold increase relative to healthy patients versus a 3.2-fold increase in non-delta, p < 0.002). After multivariable adjustment for potential confounders, miR-210 was associated with increased odds of membership in the delta subtype (aOR 1.16, 95% CI 1.05–1.29, p = 0.004) compared to non-delta sepsis. Additionally, miR-210 expression was associated with receipt of mechanical ventilation (p = 0.010), vasopressors (p = 0.032), and greater in-hospital mortality (p = 0.017).

In this prospective cohort study, circulating miR-210 was significantly increased in all subtypes of sepsis compared to control, and was further enriched in the delta sepsis subtype. After adjusting for potential confounders, including illness severity, miR-210 expression is associated with membership in the delta subtype relative to non-delta sepsis. These data suggest that activation of hypoxia-mediated pathways may contribute to different clinical subtypes of sepsis and may inform future investigation of differential treatment response.

**Figure:**
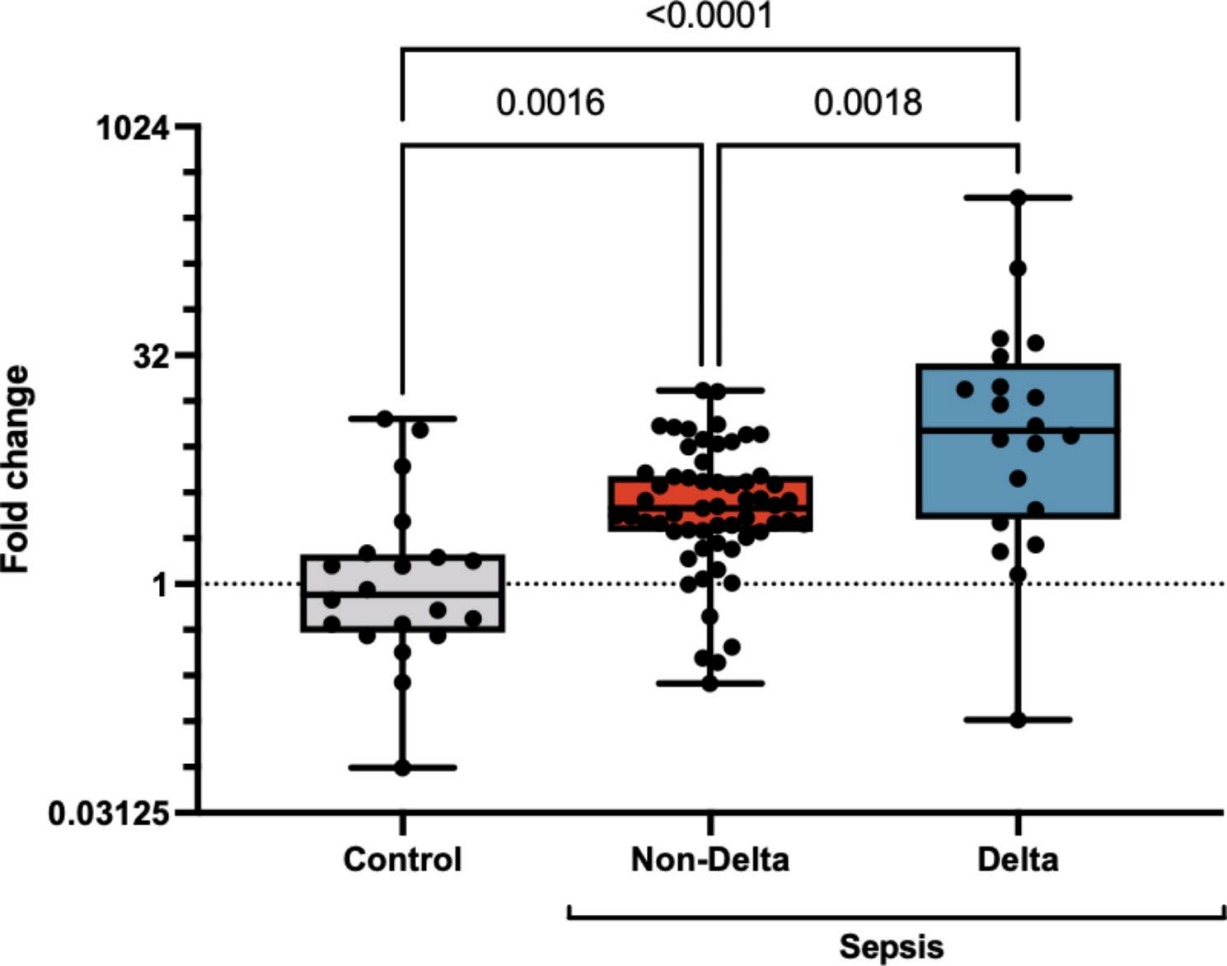
Expression of miR-210 in patients with sepsis compared to control. Expression is increased in sepsis compared to control (red and blue vs. grey) and is further enriched in the delta subtype compared to non-delta sepsis (blue vs. red). P-values represent Tukey-corrected pairwise t-tests on log_2_-transformed data.

## Electronic supplementary material

Below is the link to the electronic supplementary material.

Supplementary Material 1

Supplementary Material 2

## Data Availability

The datasets used and analyzed during the current study are available from the corresponding author on reasonable request.
