# Supplementary material for: Circulating hypoxia-dependent miR-210 is increased in clinical sepsis subtypes: A cohort study
Source: J Transl Med. 2022 Oct 4;20:448. doi: 10.1186/s12967-022-03655-6 (PMC9533507; doi:10.1186/s12967-022-03655-6)
Supplement: Supplementary file 1 — Supplementary Material 1 [file 12967_2022_3655_MOESM1_ESM.docx]

**Online Resource**

**Circulating hypoxia-dependent miR-210 is increased in clinical sepsis subtypes**

Rachel E Powell, MD^1^; Yi Yin Tai, MS^2^; Jason N Kennedy, MS^3,4^; Christopher W Seymour, MD MSc^3,4,5^; Stephen Y Chan, MD, PhD^2,*^

**Author Information**

1. Division of Pulmonary, Allergy, and Critical Care Medicine, University of Pittsburgh School of Medicine, Pittsburgh, PA, USA
2. Center for Pulmonary Vascular Biology and Medicine, Pittsburgh Heart, Lung, Blood Vascular Medicine Institute, Division of Cardiology, University of Pittsburgh School of Medicine, Pittsburgh, PA, USA
3. Clinical Research Investigation and Systems Modeling of Acute Illness (CRISMA) Center, University of Pittsburgh School of Medicine, Pittsburgh, PA, USA
4. Department of Critical Care Medicine, University of Pittsburgh School of Medicine, Pittsburgh, PA, USA
5. Department of Emergency Medicine, University of Pittsburgh School of Medicine, Pittsburgh, PA, USA

***Corresponding Author**

Stephen Y. Chan, MD, PhD

Center for Pulmonary Vascular Biology and Medicine

Pittsburgh Heart, Lung, Blood Vascular Medicine Institute

Division of Cardiology, Department of Medicine

University of Pittsburgh School of Medicine

University of Pittsburgh Medical Center

200 Lothrop Street BST E1240

Pittsburgh, PA USA 15261

Tel: 412-383-6990

Fax: 412-624-9160

Email: chansy@pitt.edu

Methods 2

Figure – Patient Accrual 4

Table 1 – Patient Characteristics 5

Table 2 – Multivariable model of delta subtype membership 6

**Methods**

This study was approved by the University of Pittsburgh Human Research Protection Office (STUDY19030218). The data were obtained under a waiver of informed consent and with authorization under the Health Insurance Portability and Accountability Act.

*Patient selection*

We chose a stratified random sample of patients from the Sepsis ENdotyping in Emergency Care (SENECA) study (NIH GM119519), which enrolled adult (age ≥ 18 years) patients meeting Sepsis-3 criteria within 6 hours of presentation to the emergency department (ED) at a tertiary care center in southwestern Pennsylvania from 2017 to 2019. We assigned patients to one of four sepsis subtypes (alpha, beta, gamma, or delta) using previously validated methods, and selected a random sample of 20 patients per subtype. A control group of 20 non-septic patients was randomly selected from the Pittsburgh Prehospital LINking Evaluation (PIPeLINE) study (NIH GM104022), which enrolled adult (age ≥18 years) non-trauma prehospital patients at risk for sepsis between August 2013 and February 2014.

*Clinical data collection*

We abstracted clinical data from the electronic health record (CERNER Co., Kansas City, MO), including patient demographics, vital signs, laboratory values, organ support, and in-hospital mortality. For descriptions of illness severity, the most abnormal vital sign and laboratory values within the first 6 hours of ED arrival were used.

*Circulating miRNA quantification*

Remnant plasma samples were collected and processed by the research team after routine clinical testing was complete. Samples were frozen at -80°C until assay. Before extraction, plasma samples were thawed and centrifuged at 12,700 rpm for 15 minutes at 4°C to remove remaining cellular debris. Circulating miRNA was extracted from 180 $\mu$L of plasma using the Qiagen miRNeasy serum/plasma kit according to the manufacturer’s instructions. To quantitatively normalize circulating miRNA plasma levels, 2 $\mu$L of 0.002 $\mu$M cel-miR-67 mimic (Thermo Fisher Scientific) was added to each 180 $\mu$L volume of plasma prior to miRNA extraction. Extracted miRNA was subsequently reverse transcribed to generate complementary DNA (cDNA) (High-Capacity cDNA Reverse Transcription Kit, Thermo Fisher Scientific) per the manufacturer’s protocol. Expression of miR-210 was evaluated using TaqMan Fast Advanced Master Mix (Thermo Fisher Scientific) suitable for the Q6 Real Time PCR device (Thermo Fisher Scientific). Expression levels were calculated using the comparative Ct method ($2^{-\Delta\Delta Ct}$) and normalized to cel-miR-67. Fold change was calculated relative to the mean Ct of the control group and was log2-transformed for statistical analysis.

*Statistical analysis*

We described demographic and clinical characteristics of patients with and without Sepsis-3. We used Fisher’s exact tests to compare categorical data. We assessed variable distributions for continuous data and used one-way ANOVA or Kruskal-Wallis tests as appropriate. We considered a two-sided p-value of 0.05 to be significant and adjusted for multiple comparisons using Tukey’s procedure.

**Figure – Patient Accrual**

**
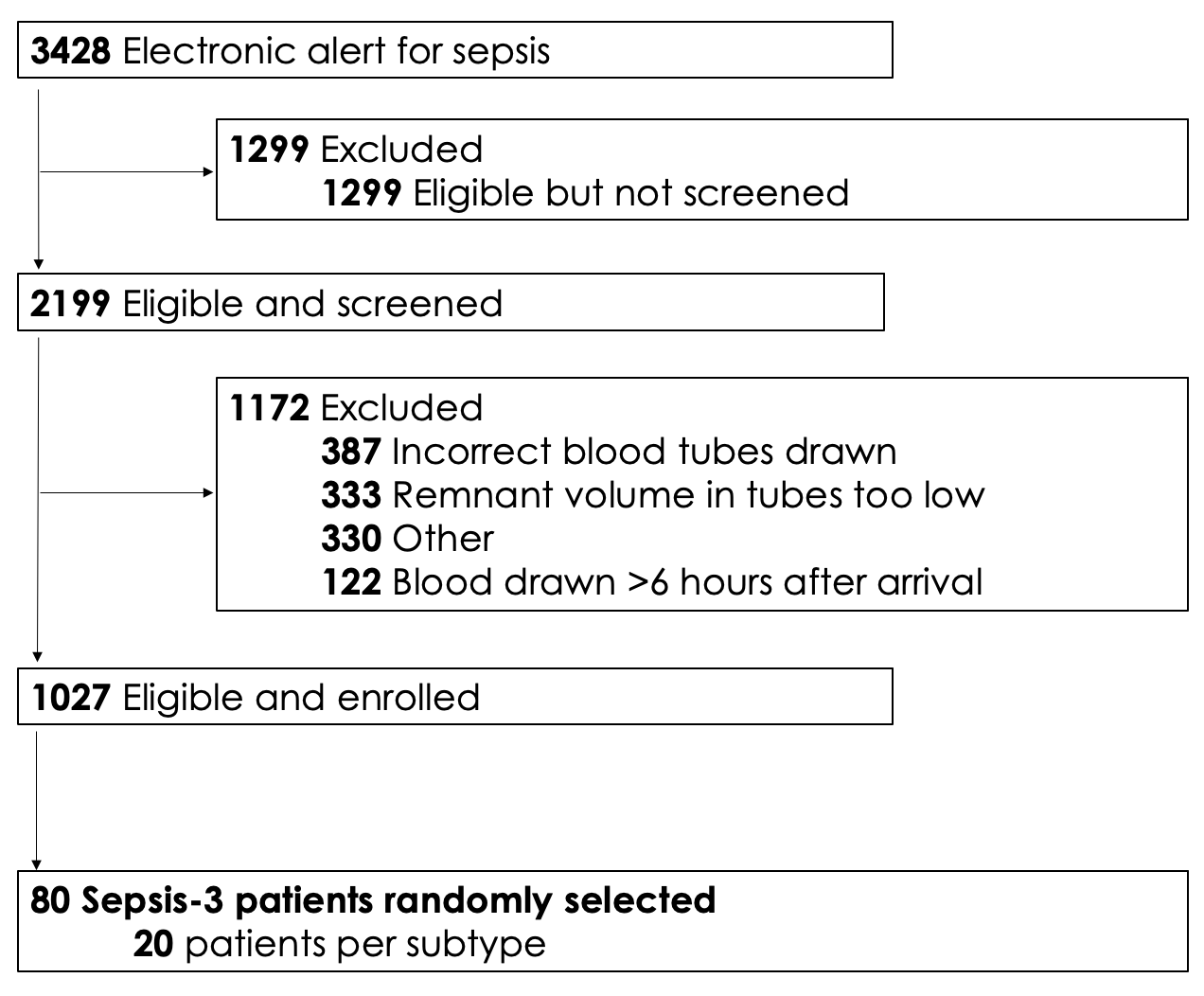
**

**Table 1 – Patient Characteristics**

|  |  | **Sepsis Subtype** | | | |
| --- | --- | --- | --- | --- | --- |
| **Characteristic** | **Control**  **(n=20)** | **Alpha**  **(n=20)** | **Beta**  **(n=20)** | **Gamma**  **(n=20)** | **Delta**  **(n=20)** |
| Age – median (IQR), years | 54 (42, 63) | 60 (43, 69) | 71 (60, 81) | 63 (43, 72) | 64 (56, 73) |
| Race/ethnicity – no. (%) |  |  |  |  |  |
| White | 9 (45) | 17 (85) | 15 (75) | 14 (70) | 13 (65) |
| Black | 11 (55) | 2 (10) | 2 (10) | 4 (20) | 4 (20) |
| Other^a^ | 0 (0) | 1 (5) | 3 (15) | 2 (10) | 3 (15) |
| Male gender – no. (%) | 9 (45) | 10 (50) | 9 (45) | 9 (45) | 13 (65) |
| Elixhauser Comorbidity Index – median (IQR)^b^ | 0 (0, 0) | 3 (2, 4) | 5 (4, 7) | 4.5 (4, 5) | 5 (3.5, 7) |
| SOFA score – median (IQR)^c, d^ | 0 (0) | 3 (2, 4) | 3 (2, 5) | 3 (3, 4) | 6 (5.5, 8) |
| Lactate – median (IQR), mmol/L^c^ | N/A ^e^ | 1.1 (0.7, 1.6) | 1.3 (0.9, 2.1) | 1.6 (1.3, 2.4) | 5.3 (2.2, 9.8) |
| ICU admission – no. (%) | 1 (5) | 9 (45) | 10 (50) | 11 (55) | 19 (95) |
| Mechanical ventilation – no. (%) | 0 (0) | 3 (15) | 1 (5) | 8 (40) | 9 (45) |
| Vasopressor use – no. (%) | 0 (0) | 0 (0) | 3 (15) | 4 (20) | 11 (55) |
| In-hospital mortality – no. (%) | 0 (0) | 1 (5) | 1 (5) | 3 (15) | 5 (25) |

^a^ Other race corresponds to Chinese, Filipino, Hawaiian, American Indian/Alaskan, Asian, Hawaiian/Other Pacific Islander, Middle Eastern, Native American, Not specified, or Pacific Islander

^b^  Elixhauser is a method of categorizing [comorbidities](http://mchp-appserv.cpe.umanitoba.ca/viewDefinition.php?definitionID=102446) of patients based on the [International Classification of Diseases](http://mchp-appserv.cpe.umanitoba.ca/viewDefinition.php?definitionID=102932) diagnosis codes found in administrative data, ranging from 0 to 31

^c^ Corresponds to maximum value within the first 6 hours

^d^ SOFA score corresponds to the severity of organ dysfunction within six hours of sepsis onset, reflecting six organ systems each with a score range of 0 to 4 points (cardiovascular, hepatic, hematologic, respiratory, neurological, renal), with a total score range of 0 to 24 points

^e^ Lactate values were missing from n=19 control patients

*Abbreviations:* IQR: interquartile range; SOFA: sequential organ failure assessment; N/A: not applicable

**Table 2 – Multivariable model of delta subtype membership (n=80)**

| **Variable** | **aOR** | **aOR 95% CI** | ***P*-value** |
| --- | --- | --- | --- |
| miR-210 fold change ^a^ | 1.16 | 1.05 – 1.29 | 0.004 |
| SOFA score in 6 hours ^b^ | 1.43 | 1.11 – 1.84 | 0.005 |
| Elixhauser ^c^ | 1.14 | 0.82 – 1.57 | 0.44 |
| Sex |  |  |  |
| Female | REF | - | - |
| Male | 1.15 | 0.28 – 4.67 | 0.85 |
| Age ^d^ | 1.02 | 0.97 – 1.06 | 0.51 |

^a^ Fold change is calculated relative to the non-septic control group. Adjusted OR corresponds to a 1 point increase in fold change.

^b^ SOFA score corresponds to the severity of organ dysfunction, reflecting six organ systems each with a score range of 0 to 4 points (cardiovascular, hepatic, hematologic, respiratory, neurological, renal), with a total score range of 0 to 24 points. Adjust OR corresponds to a 1 point change in SOFA score.

^c^ Elixhauser is a method of categorizing comorbidities of patients based on the International Classification of Diseases (ICD) diagnosis codes found in administrative data, ranging from 0 to 31. Adjusted OR corresponds to a 1 point change in Elixhauser.

^d^ Adjusted OR corresponds to a 1 year increase in age.

Abbreviations: aOR: adjusted odds ratio; CI: confidence interval; SOFA: sequential organ failure assessment score
