# Supplementary material for: Circulating hypoxia-dependent miR-210 is increased in clinical sepsis subtypes: A cohort study
Source: J Transl Med. 2022 Oct 4;20:448. doi: 10.1186/s12967-022-03655-6 (PMC9533507; doi:10.1186/s12967-022-03655-6)
Supplement: Supplementary file 2 — Supplementary Material 2 [file 12967_2022_3655_MOESM2_ESM.docx]

September 11, 2022

**Stephen Y. Chan, MD, PhD, FAHA**

*Vitalant Chair in Vascular Medicine, Professor of Medicine*

*Director, Heart, Lung, and Blood Vascular Medicine Institute*

*Director, Center for Pulmonary Vascular Biology and Medicine*

*Department of Medicine*

*University of Pittsburgh School of Medicine*

*University of Pittsburgh Medical Center*

Dr. Francesco Marincola

Editor-in-Chief

Dear Dr. Marincola,

Please find enclosed a Letter to the Editor entitled “**Circulating hypoxia-dependent miR-210 is increased in clinical sepsis subtypes**” to be considered for publication in *Journal of Translational Medicine****.***

Based on our prior collective work, patients with community-onset sepsis can be grouped into 4 distinct subtypes (alpha, beta, gamma, and delta) based on routinely available clinical data. These subtypes differ both in outcome and response to treatment, though the biologic mechanisms underlying these differences are unknown. Because ischemic end-organ damage and endothelial dysfunction may contribute to differences in sepsis, we hypothesized that treatment-response subtypes may be partially explained by differential activation of hypoxia-mediated pathways. Multiple studies show that circulating microRNAs (miRNAs) are candidate biomarkers for acute illness, but research in sepsis is limited. As a ubiquitously expressed miRNA that regulates key downstream responses to hypoxia and can be delivered to the endothelium as a biologically active molecule from the circulating bloodstream, miR-210 is a promising candidate to study in sepsis. In this prospective cohort study, circulating miR-210 was significantly increased in all subtypes of sepsis compared to control, and was further enriched in the delta sepsis subtype. These data suggest that activation of hypoxia-mediated pathways may contribute to different clinical subtypes of sepsis and may inform future investigation of differential treatment response.

Conflicts of interest include that I, Stephen Y. Chan, have served as a consultant for Acceleron Pharma and United Therapeutics and have held research grants from Actelion, Bayer, and Pfizer. I additionally am a director, officer, and shareholder of Synhale Therapeutics. I have filed patents regarding metabolic dysregulation in pulmonary hypertension. The other authors declare no competing interests. These conflicts of interest are additionally reported in the manuscript.

A subset of this work was presented at the American Thoracic Society 2022. Otherwise, this manuscript has not been published in print or electronic format, and it is not under consideration by another publication or electronic medium. All authors have agreed to the content of the letter and submission for publication in *Journal of Translational Medicine*. Please let me know if you have any questions or require any additional information. Thank you for your consideration.

Sincerely,

Stephen Y. Chan, MD, PhD
